# The efficacy and safety of pre-hospital cooling after out-of-hospital cardiac arrest: a systematic review and meta-analysis

**DOI:** 10.1186/s13054-018-1984-2

**Published:** 2018-03-13

**Authors:** Patrick J. Lindsay, Danielle Buell, Damon C. Scales

**Affiliations:** 1 Department of Internal Medicine, University of Toronto, Toronto, ON Canada; 2 Department of Critical Care Medicine, Sunnybrook Health Sciences Centre, Toronto, ON Canada; 3 Interdepartmental Division of Critical Care Medicine, University of Toronto, Toronto, ON Canada

**Keywords:** Therapeutic hypothermia, Out-of-hospital cardiac arrest, Pre-hospital, Cooling

## Abstract

**Background:**

Mild therapeutic hypothermia (TH), or targeted temperature management, improves survival and neurological outcomes in patients after out-of-hospital cardiac arrest (OHCA). International guidelines strongly support initiating TH for all eligible individuals presenting with OHCA; however, the timing of cooling initiation remains uncertain. This systematic review and meta-analysis was conducted with all available randomised controlled trials (RCTs) included to explore the efficacy and safety of initiating pre-hospital TH in patients with OHCA.

**Methods:**

The MEDLINE and Cochrane databases were searched from inception to October 2017. Inclusion criteria for full-text review included RCTs comparing pre-hospital TH with no pre-hospital TH after cardiac arrest, patients > 14 years of age with documented cardiac arrest from any rhythm, and outcome data that included survival to hospital discharge and temperature at hospital arrival. Results of retrieved studies were compared through meta-analysis using random effects modelling.

**Results:**

A total of 10 trials comprising 4220 patients were included. There were no significant differences between the two arms for the primary outcome of neurological recovery (risk ratio [RR] 1.04, 95% CI 0.93–1.15) or the secondary outcome of survival to hospital discharge (RR 1.01, 95% CI 0.92–1.11). However, there was a significantly lower temperature at hospital arrival in patients receiving pre-hospital TH (mean difference − 0.83, 95% CI − 1.03 to − 0.63). Pre-hospital TH significantly increased the risk of re-arrest (RR 1.19, 95% CI 1.00 to 1.41). No survival differences were observed among subgroups of patients who received intra-arrest TH vs post-arrest TH or who had shockable vs non-shockable rhythms.

**Conclusions:**

Pre-hospital TH after OHCA effectively decreases body temperature at the time of hospital arrival. However, it does not improve rates of survival with good neurological outcome or overall survival and is associated with increased rates of re-arrest.

**Electronic supplementary material:**

The online version of this article (10.1186/s13054-018-1984-2) contains supplementary material, which is available to authorized users.

## Background

Targeted temperature management (TTM) or mild therapeutic hypothermia (TH) has been shown to improve survival and neurological outcomes in patients after out-of-hospital cardiac arrest (OHCA) [1]. Compared with no treatment, cooling the body to 32–34 °C leads to an estimated 35% relative increase in survival [2]. More recent research suggests that cooling to 36 °C results in benefits similar to cooling to 32–34 °C [3]. International guidelines strongly support initiating TH for all eligible individuals presenting with OHCA, but they acknowledge that the optimal target temperature and timing of cooling initiation remain uncertain [4, 5]. Notably, observational studies and secondary outcomes suggest improved neurological outcomes and survival with earlier and more rapid initiation of cooling, such as initiating cooling prior to hospital arrival [6–8].

Multiple randomised controlled trial (RCTs) have investigated the safety and efficacy of pre-hospital TH; however, all have failed to provide strong evidence to support its widespread adoption. The lack of persuasive data could be attributed to underpowered studies, heterogeneity in protocols (e.g., cooling methods, intra-arrest vs post-arrest) and the widespread implementation of TH at accepting institutions. Previous meta-analyses have also failed to provide strong data to support recommendations, but these did not include the most recent large trials of pre-hospital cooling [9–12]. We therefore conducted a systematic review and meta-analysis including all available RCTs to explore the efficacy and safety of pre-hospital TH in patients with OHCA.

## Methods

This systematic review and meta-analysis conformed to the Preferred Reporting Items for Systematic Reviews and Meta-Analyses (PRISMA) statement [13].

### Data search

The online search strategy used both the MEDLINE and Cochrane Library databases from inception until October 2017. The following terms were used: “out-of-hospital cardiac arrest” or “heart arrest” or “cardiac arrest” or “death, sudden” or “ventricular fibrillation” or “pulseless electrical activity” or “PEA” or “asystole” or “tachycardia” and “cryotherapy” or “hypothermia, induced” or “hypothermia” or “cooling” or “targeted temperature management or TTM” and “emergency medical services” or “emergency responders” or “emergency medical technicians” or “paramedic” or “prehospital” or “advanced life support” or “out of hospital.” Additionally, we checked reference lists of relevant studies and review articles.

### Study selection

Retrieved abstracts were assessed by two reviewers (PJL and DB) to evaluate whether they met the following inclusion criteria for full-text review: (1) RCT evaluating pre-hospital TH vs no pre-hospital TH after cardiac arrest; (2) patients > 14 years of age; (3) patients with documented cardiac arrest from rhythms, including ventricular fibrillation (VF), ventricular tachycardia, pulseless electrical activity and asystole; and (4) outcome data that included survival to hospital discharge and temperature at hospital arrival. The same two reviewers completed full-text reviews to identify included studies. A third reviewer resolved any disagreements.

### Data extraction

Two authors (DB and PJL) extracted the following data independently using a standard data extraction form: publication year, study design, study population characteristics, initial cardiac rhythm, timing of cooling, cooling procedures, primary and secondary outcome measures, and study quality.

### Risk-of-bias assessment

Study quality was appraised using the Cochrane Risk of Bias Tool for RCTs [13]. The assessment includes evaluation of random sequence generation, allocation concealment, blinding, incomplete outcome data and selective outcome reporting.

### Study outcome definition

The primary outcome of this systematic review was survival to hospital discharge with a favourable neurological outcome. Favourable neurological outcome was defined as a the patient discharged to home or to rehabilitation, Cerebral Performance Categories Scale (CPC) score of 1 or 2 or a modified Rankin Scale score of 0, 1 or 2 [14, 15]. Secondary outcomes were survival to hospital discharge and temperature upon hospital admission. The safety outcomes included pulmonary oedema and recurrent arrest during transport to the hospital.

### Data synthesis and analysis

We conducted a meta-analysis of results of the included studies using Review Manager software version 5.3. We summarized categorical data using the risk ratio (RR) according to the Mantel-Haenszel method and a random effects model [16]. For continuous data, we estimated the mean difference (MD) using the inverse variance method and fixed effects. Heterogeneity was detected with a chi-square test with *n* − 1 degrees of freedom, which was expressed as *I*^2^. When the *I*^2^ statistic was > 50, statistical heterogeneity was considered to be relevant. Sensitivity analysis were performed to further explore heterogeneity by excluding one study at a time, deleting studies with excessively high weights in pooled studies, and excluding studies that used discharge destination as a surrogate for neurological outcome.

## Results

### Search results and study selection

**Fig. 1:**
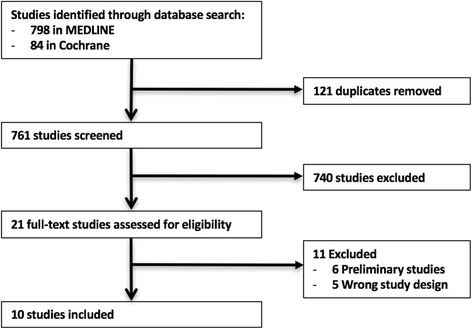
Flow diagram of study selection

Our search strategy yielded 798 citations in MEDLINE and 84 citations in the Cochrane database, from which 121 duplicates were removed, leaving 761 studies to be screened. Of these, 21 full texts were reviewed, with 10 meeting the study inclusion criteria. All ten were included in the systematic review (Fig. 1).

### Characteristics of studies

Table 1 demonstrates the characteristics of the ten included studies, all of which were RCTs published between 2007 and 2017. Nine were single-country trials, and one was a multi-country trial. Of the single-country trials, four were conducted in Australia [6, 11, 17, 18], two in the United States [19, 20], one in Canada [12], one in Finland [21] and one in France [22]. The multi-country trial included five European countries [23]. Seven of the ten studies included patients with any initial cardiac rhythm. The remaining three studies included only patients with VF [6, 17] and non-shockable rhythms [18], respectively, as the initial cardiac rhythm. TH was initiated after return of spontaneous circulation (ROSC) in seven studies [6, 12, 17–21] and intra-arrest in three [11, 22, 23]. The majority of studies reported cooling the patients in the pre-hospital TH arm using surface cooling measures [12, 17–19, 22], an infusion of a cold solution, normal saline or Ringer’s lactate [11, 12, 17–22], and one used trans-nasal evaporative cooling [23]. One study used only ice packs applied to the patient’s head and torso [6]. All studies reported survival at hospital discharge and neurological status at discharge, and all studies reported temperature at time of admission to hospital. A summary of the results of the studies is provided in Table 2.

**Table 1:** Characteristics of included studies

First author, publication year (site) [reference]	Timing of intervention	Primary cardiac rhythm	Cooling method (intervention)	Cooling methods in-hospital (intervention/control)		Outcome (efficacy and safety)		Outcome (pre-hospital safety)
Bernard, 2002 (Australia) [6]	Post-arrest	VF	Application of ice packs to patient’s head and torso	Application of ice packs to patient’s head, neck, torso and limbs. When 33 °C temperature was achieved, ice packs were removed	No cooling implemented pre-hospital or in-hospital	Discharged directly to home or to a rehabilitation facility	Survival/favourable outcome at discharge^a^	N/A
Bernard, 2010 (Australia) [17]	Post-arrest	VF	Infusion of up to 2 L of ice-cold lactated Ringer’s solution commenced at 100 ml/minute	Additional 10–20 ml/kg rapid infusion of ice-cold Ringer’s lactate, then surface cooling pads	Rapid infusion of 40 ml/kg of ice-cold Ringer’s lactate, then surface cooling pads	Discharged directly to home or to a rehabilitation facility	Survival/favourable outcome at discharge, temperature at admission^a^	Pulmonary oedema
Bernard, 2012 (Australia) [18]	Post-arrest	Asystole/PEA	Cooled intravenous fluids, ice packs and cooling blankets	Additional 40 ml/kg rapid infusion of ice-cold Hartmann’s solution, then surface cooling pads	40 ml/kg rapid infusion of ice-cold Hartmann’s solution, then surface cooling pads	Discharged directly to home or to a rehabilitation facility	Survival/favourable outcome at discharge, temperature at admission, pre-hospital survival^a^	Pulmonary oedema
Bernard, 2016 (Australia) [11]	Intra-arrest	VF, VT, Asystole, PEA	Infusion of 30 ml/kg cold saline (maximum 2 L)	N/A	N/A	Discharged directly to home or to a rehabilitation facility	Survival at hospital discharge, discharge to home from hospital, proportion of patients in shockable and non-shockable rhythms with ROSC^a^	Pulmonary oedema
Castren, 2010 (multi-site) [23]	Intra-arrest	VF, VT, Asystole, PEA	Trans-nasal evaporative cooling	Cooled in hospital according to institutional standards	Cooled in-hospital according to institutional standards	CPC score 1 or 2	Safety and efficacy of RhinoChill intra-nasal cooling system (BeneChill, San Diego, CA, USA), temperature at admission, ROSC, survival at discharge, neurological function^a^	Pulmonary oedema, re-arrest, bleeding
Debaty, 2014 (France) [22]	Intra-arrest	VF, VT, PEA, Asystole	Up to 2000 ml of ice-cold 0.9% saline solution at 100 ml/minute, then surface cooling using gel pads	Cooling continued with cold saline infusion, cooling mattress, cold air circulation and/or extracorporeal life support	Cooled with cold saline infusion, cooling mattress, cold air circulation and/or extracorporeal life support	CPC score 1 or 2	Temperature at admission, ROSC, survival and neurological function (discharge/30 days/1 year)	Pulmonary oedema, bleeding, infection, arrhythmia
Kamarainen, 2009 (Finland) [21]	Post-arrest	VF, VT, PEA, Asystole	4 °C Ringer’s acetate at approximately 100 ml/minute	Cooling continued at the discretion of hospital physicians	Cooling initiated at the discretion of hospital physicians	CPC score 1 or 2	Temperature at admission, survival at discharge, neurological function^a^	Pulmonary oedema, re-arrest
Kim, 2007 (United States) [20]	Post-arrest	VF, VT, PEA, Asystole	Up to 2 L of 4 °C normal saline solution	According to physician preferences	According to physician preferences	Absence of severe neurological deficit (undefined)	Temperature at admission, survival at discharge^a^	Pulmonary oedema, re-arrest
Kim, 2014 (United States) [19]	Post-arrest	VF, VT, PEA, Asystole	Up to 2 L of 4 °C normal saline solution	Surface and intravascular cooling	Surface and intravascular cooling	Full neurological recovery/mild impairment	Survival at discharge,^a^ neurological function^a^ and temperature at admission	Pulmonary oedema, re-arrest
Scales, 2017 (Canada) [12]	Post-arrest	VF, VT, PEA, Asystole	Application of ice packs to neck, axillae and groins, and infusion of up to 2 L of cold saline (0.9% sodium chloride solution at approximately 4 °C)	According to physician preferences	According to physician preferences	mRS 0, 1 or 2	Successful TTM,^a^ survival to hospital discharge, good neurological outcome, temperature at admission	Pulmonary oedema, re-arrest

*Abbreviations: CPC* Cerebral Performance Categories Scale; *mRS* Modified Rankin Scale, *PEA* Pulseless electrical activity, *ROSC* Return of spontaneous circulation, *TTM* Targeted temperature management, *VF* Ventricular fibrillation, *VT* Ventricular tachycardia

^a^Primary outcome of study

**Table 2:** Outcome data from included studies

First author, year (site) [reference]	Number of participants (total/I/C)	Survival to discharge (I vs C)		Survival of those with shockable rhythm (I vs C)		Temperature at hospital arrival (I vs C)		Pulmonary oedema (I vs C)		Re-arrest (I vs C)		Mean pre-hospital volume infused (ml)	Favourable neurological outcome (I vs C)		Survival at hospital arrival (I vs C)	
Bernard, 2002 (Australia) [6]	77/43/34	21 (49%)	11 (32%)	N/A	N/A	35.0	35.5	N/A	N/A	N/A	N/A	N/A	21 (49%)	9 (26%)	N/A	N/A
Bernard, 2010 (Australia) [17]	234/118/116	56 (47%)	62 (53%)	N/A	N/A	34.6	35.4	0	0	0	0	1900	56 (48%)	61 (53%)	N/A	N/A
Bernard, 2012 (Australia) [18]	163/82/81	11 (13%)	7 (9%)	N/A	N/A	34.4	35.7	0	0	N/A	N/A	1500	10 (12%)	7 (10%)	75 (91%)	74 (91%)
Bernard, 2016 (Australia) [11]	1198/618/580	63 (10%)	66 (11%)	55 (19%)	62 (23%)	34.7	35.4	62 (10%)	26 (45%)	N/A	N/A	1200	63 (10%)	63 (11%)	304 (49%)	317 (55%)
Castren, 2010 (multi-site) [23]	200/96/104	14 (15%)	13 (13%)	10 (37%)	10 (33%)	34.2	35.5	0	0	3 (3%)	2 (2%)	N/A	11 (11%)	9 (9%)	32 (33%)	42 (41%)
Debaty, 2014 (France) [22]	245/123/122	7 (6%)	5 (4%)	N/A	N/A	33.9	35	7 (6%)	8 (7%)	N/A	N/A	1000	7 (6%)	4 (3%)	41 (33%)	36 (30%)
Kamarainen, 2009 (Finland) [21]	37/19/18	11 (58%)	10 (56%)	N/A	N/A	34.1	35.2	0	0	2 (11%)	3 (17%)	2370	8 (42%)	8 (44%)	N/A	N/A
Kim, 2007 (United States) [20]	125/63/62	21 (67%)	18 (71%)	19 (66%)	10 (45%)	34.7	35.7	24 (38%)	27 (44%)	15 (24%)	13 (21%)	1236	19 (30%)	16 (26%)	49 (78%)	48 (77%)
Kim, 2014 (United States) [19]	1359/688/671	259 (38%)	249 (37%)	183 (63%)	187 (64%)	34.8	35.8	256 (37%)	184 (27%)	176 (26%)	138 (21%)	1435	231 (34%)	225 (33%)	679 (99%)	660 (98%)
Scales, 2017 (Canada) [12]	582/279/303	92 (33%)	98 (31%)	79 (64%)	74 (55%)	35.1	35.2	33 (12%)	54 (18%)	21 (8%)	25 (8%)	640	82 (29%)	76 (26%)	250 (90%)	258 (85%)

*I* Intervention (pre-hospital therapeutic hypothermia), *C* Comparator

### Quality of included studies

Overall, the risk of bias for the included studies was low (Additional file 1: Figures S1 and S2). A potential for selection bias was noted in one study owing to randomization according to day of the month and possible unmasking of allocation concealment [6]. Across the studies, the nature of the intervention made true blinding of the providers impossible. Additionally, in one study, leaving the receiving practitioner unblinded was an intended intervention, because the authors postulated that this would reduce the time to in-hospital cooling [12]. Outcome assessors for the primary endpoints were blinded in eight of the ten studies. In all of the studies except two, researchers performed an intention-to-treat analysis [6, 21]. All studies had concerns for other sources of bias: Four studies were terminated before the target sample size was recruited [11, 12, 17, 18]; one study was funded by a company with an invested interest [23]; and two studies had concerns for possible selection bias, with 7, 497 and 23 patients simply missed and not included in the study [6, 19, 20].

### Effects of interventions

#### Neurological function

**Fig. 2:**
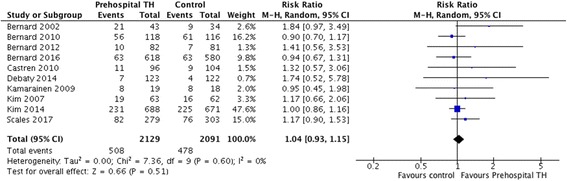
Risk ratio of favourable neurological outcome. *M-H* Mantel-Haenszel method, *TH* Therapeutic hypothermia

All included studies reported neurological outcomes at hospital discharge (2129 cases and 2091 control subjects). No differences were observed in rates of favourable neurological outcome at hospital discharge between the pre-hospital TH arm and the control arm (RR 1.04, 95% CI 0.93 to 1.15, *I*^2^ = 0%) (Fig. 2). Excluding trials that used discharge destination as a surrogate for neurological outcome produced similar results (RR 1.18, 95% CI 0.93 to 1.49, *I*^2^ = 0%) (Additional file 1: Figure S3).

#### Survival to hospital discharge

**Fig. 3:**
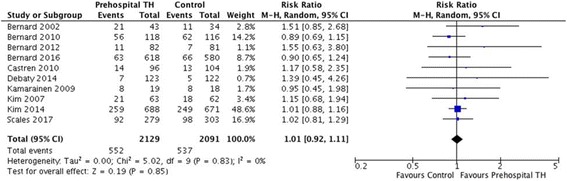
Risk ratio of survival at discharge. *M-H* Mantel-Haenszel method, *TH* Therapeutic hypothermia

All ten studies reported rates of survival to hospital discharge (2129 cases and 2091 controls). The pooled survival rate was similar when we compared the pre-hospital TH arm with the control arm (RR 1.01, 95% CI 0.92 to 1.11, *I*^2^ = 0) (Fig. 3). Sensitivity analyses using data from trials (*n* = 7) that stratified patients according to initial rhythm (i.e., shockable vs non-shockable) [6, 11, 12, 17, 19, 20, 23] also showed no effect of pre-hospital TH on survival to hospital discharge (Additional file 1: Figures S4 and S5).

#### Temperature at admission

**Fig. 4:**
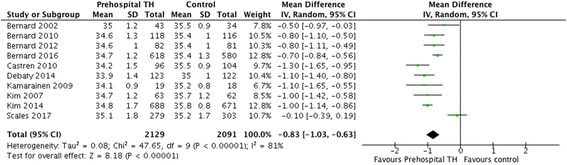
Mean temperature difference upon hospital arrival. *TH* Therapeutic hypothermia

All studies reported temperature upon hospital arrival, and overall there was a significantly lower temperature at time of admission for those in the pre-hospital TH arm than in the control arm (MD = − 0.83, 95% CI − 1.03 to − 0.63, *I*^2^ = 81%) (Fig. 4). A sensitivity analysis removing one trial with discordant findings reduced heterogeneity but produced similar results (MD = − 0.91; 95% CI − 1.06 to − 0.76, *I*^2^ = 61%) (Additional file 1: Figure S6).

#### Pre-hospital pulmonary oedema and re-arrest

Researchers in all studies with the exception of one [6] evaluated their patients for pulmonary oedema. The studies relied on chest x-ray findings or froth visible in endotracheal tubes; however, none of the researchers reported using explicit criteria for pulmonary oedema diagnosis. No differences between groups were observed in the pooled results, but there was significant heterogeneity (RR 1.12, 95% CI 0.75 to 1.67, *I*^2^ = 80%) (Additional file 1: Figure S7).

**Fig. 5:**
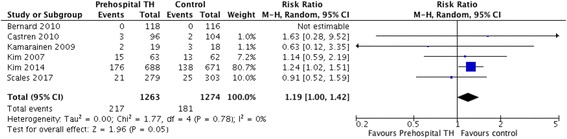
Risk ratio of re-arrest. *M-H* Mantel-Haenszel method, *TH* Therapeutic hypothermia

Rates of re-arrest after ROSC (*n* = 6 trials, comprising 1263 cases and 1274 control subjects) were higher among patients treated with pre-hospital TH (RR 1.19, 95% CI 1.00 to 1.41, *I*^2^ = 0%) (Fig. 5). In contrast, there were no significant differences between the two arms in rates of survival to hospital admission (RR 1.00, 95% CI 0.94 to 1.05, *n* = 7 trials comprising 1949 cases and 1923 control subjects), although greater heterogeneity was observed for this outcome (*I*^2^ = 60%) (Additional file 1: Figure S8) [11, 12, 18–20, 22, 23].

#### Subgroup analyses

Pre-specified subgroup analyses were performed to evaluate whether initiation of cooling during the arrest (*n* = 3 trials) vs initiation of cooling after ROSC (*n* = 7 trials) led to differences in clinical outcomes, including survival to hospital discharge and rate of re-arrest. No differences in rates of survival to hospital discharge (Additional file 1: Figure S9) or rates of re-arrest (Additional file 1: Figure S10) were observed across these subgroups.

## Discussion

Our systematic review and meta-analysis of pre-hospital cooling after cardiac arrest is the largest to date, to our knowledge, comprising 4220 patients from 10 trials. Our analysis shows that pre-hospital induction of mild TH reduces the temperature at hospital arrival but does not improve overall survival or survival with good neurological outcome. These results were consistent among patients with shockable and non-shockable initial cardiac rhythms and did not vary according to the timing of cooling initiation (i.e., intra-arrest vs after ROSC).

Previous meta-analyses have evaluated pre-hospital TH after cardiac arrest, but ours is the first, to our knowledge, to include all available trials, resulting in nearly double the total sample size of previous reviews [9, 10, 24–26]. Despite the improved power and precision of our review, we still detected no benefit of pre-hospital TH in our primary outcome of survival with good neurological outcome. A limitation of included studies relates to the inconsistent definition of good neurological outcome, however. For example, researchers in one trial reported only rates of broadly defined ‘severe neurological deficits’ [20]. In three of the included studies, investigators reported rates of discharge to home or to a rehabilitation facility as a surrogate for good neurological outcome [11, 17, 18]; yet, it was unclear whether any of the patients were discharged to home with only palliative care services. Researchers in one study reported good neurological outcome as the absence of neurological deficit. The remaining studies used an objective measure of neurological outcome with the CPC and modified Rankin Scale scores or full neurological recovery, which allows for more consistent comparisons [12, 19, 21–23].

Similar to earlier studies, our review suggests that pre-hospital TH may increase the rate of re-arrest [10, 26]. This result was strongly influenced by one trial in which the re-arrest rate was significantly higher than in the control group [19]. The authors of that study postulated that rapid fluid infusion resulted in volume overload, which in the context of resuscitation may have led to higher re-arrest rates. However, we observed substantial heterogeneity across studies for this finding, and the higher re-arrest rate did not result in different rates of survival to hospital admission. It has been suggested that very early cooling—during the arrest or immediately following ROSC—may also increase re-arrest risk, but our subgroup analysis did not support this hypothesis. There were no differences between groups in rates of pulmonary oedema, but this outcome was characterized by marked heterogeneity, and none of the studies documented explicit criteria for systematic screening for pulmonary oedema.

Our review is limited by methodological heterogeneity across all of the included papers. Although the majority of studies in this review were focused on measuring the same primary and secondary outcomes, there were a variety of differences in the protocols followed once patients were admitted to hospital, with in-hospital cooling typically left to the discretion of the unblinded treating physician. In-hospital cooling methods ranged from ice-cold intravenous fluids to surface cooling to not being cooled at all. Other sources of treatment heterogeneity arise from ongoing debate surrounding the optimal temperature target and duration of cooling, as well as practice pattern variability for timing of withdrawal of life-sustaining therapy, all of which could impact outcomes [3, 27, 28]. Further research should be done to investigate the effect of pre-hospital TH in the setting of a more standardized approach to in-hospital post-resuscitation care. Other limitations of studies included in our review include a lack of blinding of care providers, which may have introduced bias if other treatments were altered as a result of knowledge of treatment allocation, and possible selection bias.

## Conclusions

Our review demonstrates that pre-hospital TH after OHCA effectively decreases body temperature at time of hospital arrival, but it does not improve rates of survival with good neurological outcome or overall survival. Furthermore, this study illustrates that there may be increased risk of adverse outcomes, with rates of re-arrest higher in pre-hospital TH.

## Additional files

Additional file 1: **Figure S1.** Risk-of-bias graph: Review authors’ judgements about each risk-of-bias item. **Figure S2.** Risk-of-bias summary: review authors’ judgements about each risk of bias for each included study. **Figure S3.** Risk ratio of favourable neurological outcome sensitivity analysis. **Figure S4.** Risk ratio of survival to discharge with a shockable VF rhythm. **Figure S5.** Risk ratio of survival to discharge with a non-shockable rhythm. **Figure S6.** Sensitivity analysis of temperature upon hospital admission with Scales et al. [12] study removed. **Figure S7.** Risk ratio of pulmonary oedema. **Figure S8.** Risk ratio of survival at hospital arrival. **Figure S9.** Subgroup analysis for timing of cooling and survival to discharge. **Figure S10.** Subgroup analysis for timing of cooling and re-arrest. (DOCX 9669 kb)

## Abbreviations

C: Comparator
CPC: Cerebral Performance Categories Scale
I: Intervention
MD: Mean difference
M-H: Mantel-Haenszel method
mRS: Modified Rankin Scale
OHCA: Out-of-hospital cardiac arrest
PEA: Pulseless electrical activity
PRISMA: Preferred Reporting Items for Systematic Reviews and Meta-Analyses statement
RCT: Randomised controlled trial
ROSC: Return of spontaneous circulation
RR: Risk ratio
TH: Therapeutic hypothermia
TTM: Targeted temperature management
VF: Ventricular fibrillation
VT: Ventricular tachycardia
