# Supplementary material for: The efficacy and safety of pre-hospital cooling after out-of-hospital cardiac arrest: a systematic review and meta-analysis
Source: Crit Care. 2018 Mar 13;22:66. doi: 10.1186/s13054-018-1984-2 (PMC5850970; doi:10.1186/s13054-018-1984-2)
Supplement: Supplementary file 1 — Figure S1. Risk-of-bias graph: Review authors’ judgements about each risk-of-bias item. Figure S2. Risk-of-bias summary: review authors’ judgements about each risk of bias for each included study. Figure S3. Risk ratio of favourable neurological outcome sensitivity analysis. Figure S4. Risk ratio of survival to discharge with a shockable VF rhythm. Figure S5. Risk ratio of survival to discharge with a non-shockable rhythm. Figure S6. Sensitivity analysis of temperature upon hospital admission with Scales et al. [12] study removed. Figure S7. Risk ratio of pulmonary oedema. Figure S8. Risk ratio of survival at hospital arrival. Figure S9. Subgroup analysis for timing of cooling and survival to discharge. Figure S10. Subgroup analysis for timing of cooling and re-arrest. (DOCX 9669 kb) [file 13054_2018_1984_MOESM1_ESM.docx]

**Additional file 1**


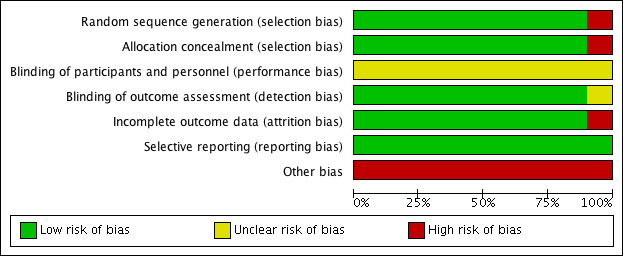


Figure S1: Risk of bias graph: Review authors judgements about each risk of bias item


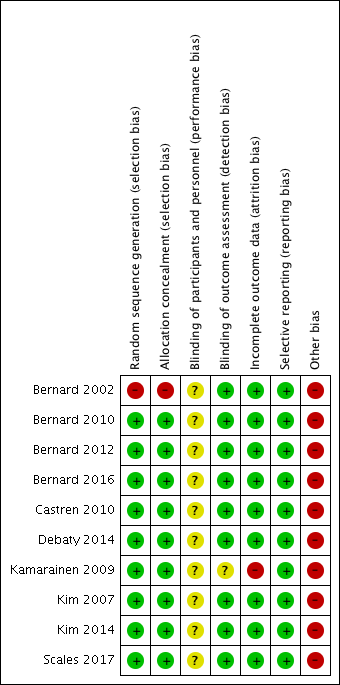


Figure S2: Risk of bias summary: review authors judgements about each risk of bias for each included study


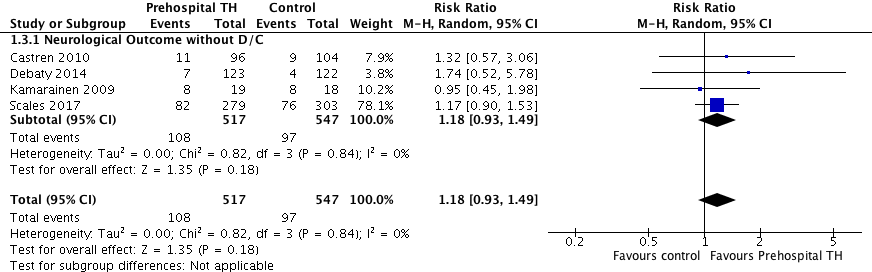


Figure S3: Risk ratio of favorable neurological outcome sensitivity analysis


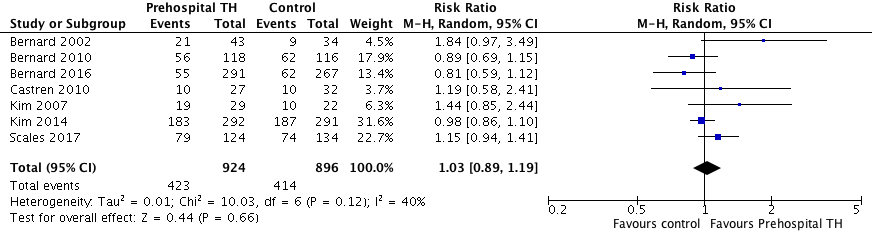


Figure S4: Risk ratio of survival to discharge with a shockable/VF rhythm


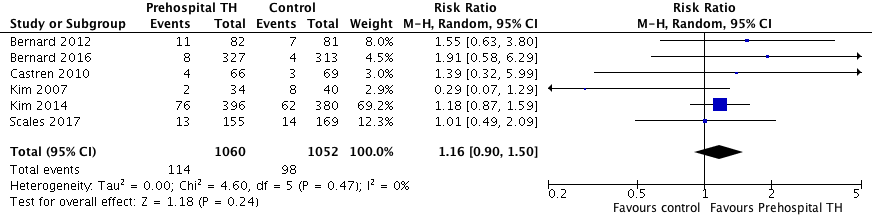


Figure S5: Risk ratio of survival to discharge with a non-shockable rhythm


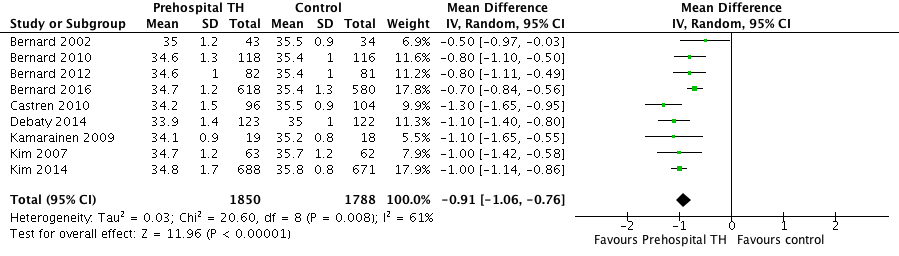


Figure S6: Sensitivity analysis of temperature upon hospital admission with Scales et al removed


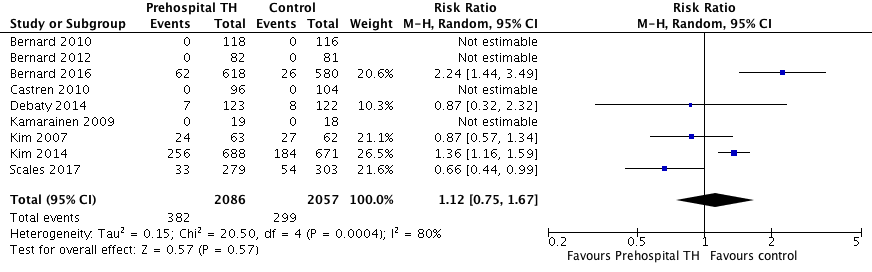


Figure S7: Risk ratio of pulmonary edema


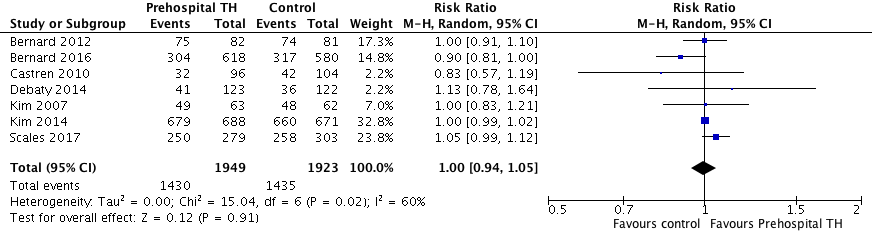


Figure S8: Risk ratio survival at hospital arrival


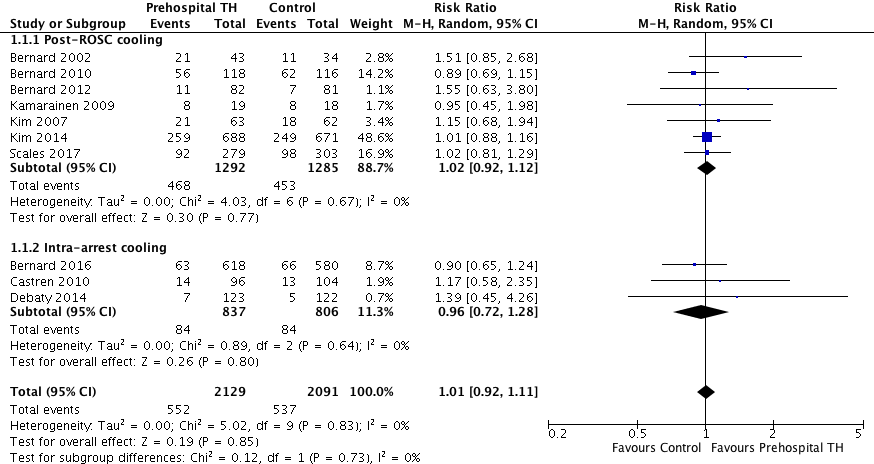


Figure S9: Subgroup analysis for timing of cooling and survival to discharge


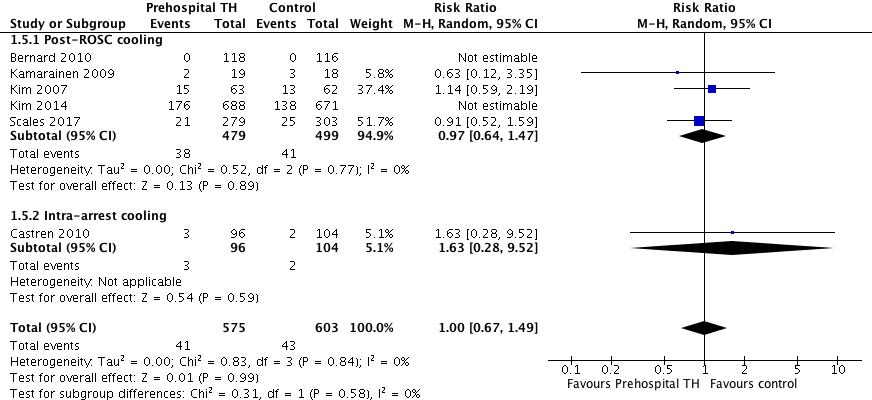


Figure S10: Subgroup analysis for timing of cooling and rearrest
